# Quantitative Research of ^11^C-CFT and ^18^F-FDG PET in Parkinson’s Disease: A Pilot Study With NeuroQ Software

**DOI:** 10.3389/fnins.2019.00299

**Published:** 2019-04-05

**Authors:** Xun Sun, Fang Liu, Qingyao Liu, Yongkang Gai, Weiwei Ruan, Dilani Neranjana Wimalarathne, Fan Hu, Xubo Tan, Xiaoli Lan

**Affiliations:** ^1^Department of Nuclear Medicine, Union Hospital, Tongji Medical College, Huazhong University of Science and Technology, Wuhan, China; ^2^Hubei Key Laboratory of Molecular Imaging, Wuhan, China

**Keywords:** Parkinson’s disease, ^11^C-CFT, ^18^F-FDG, PET, diagnosis, NeuroQ

## Abstract

Dopamine transporter (DAT) and glucose metabolism imaging have been applied in the diagnosis of Parkinson’s disease (PD). We explored the possibility of evaluating for PD with NeuroQ software by analyzing ^11^C-2β-carbomethoxy-3β-(4-fluorophenyl) tropane (^11^C-CFT) and ^18^F-FDG PET/CT. We retrospectively analyzed brain ^11^C-CFT and ^18^F-FDG PET/CT of 38 patients with parkinsonism, including 20 with PD, 10 with multiple system atrophy (MSA) and 8 with essential tremor (ET), and compared them with the PET/CT of 11 normal healthy controls (NC). PD patients were divided into mild and moderate-severe grade according to the Hoehn-Yahr (H&Y) scale. The ^11^C-CFT uptake in the caudate nuclei (CN) and putamen (Pu) normalized with cerebellum (CN/Cb and Pu/Cb) were obtained with a manual method and NeuroQ software, and their diagnostic performance was compared.^18^F-FDG uptake of specific regions was also obtained with NeuroQ, and the enhancement effect for the differential diagnosis was evaluated. There was significant agreement between the manual method and the NeuroQ method for ^11^C-CFT uptake by CN (*r^2^*= 0.680) and Pu (*r^2^*= 0.770). ^11^C-CFT uptake by CN and Pu in PD and MSA patients was significantly lower compared to NC and ET patients. The cutoffs of CN/Cb and Pu/Cb for the distinction between PD and NC were 1.71 and 2.20, respectively. No difference in uptake ratios occurred between PD and MSA. ^18^F-FDG uptake by the pons and cerebellum in the MSA group was markedly decreased. It was highly accurate in distinguishing between PD and MSA when combined with analysis of ^11^C-CFT uptake. Pu/Cb decreased significantly in mild grade PD compared to NC group (1.92 ± 0.33 vs. 2.82 ± 0.43); however no statistically significant decrease in CN/Cb was observed until moderate-severe grade PD (1.43 ± 0.11 vs. 2.23 ± 0.36). In early asymmetric PD, a statistically significant difference could be seen with Pu/Cb between the symptomatic and asymptomatic side (2.17 ± 0.30 vs. 1.95 ± 0.22). ^11^C-CFT and ^18^F-FDG PET/CT can be analyzed quantitatively with NeuroQ software, which provides an accurate method for the diagnosis and severity evaluation of PD.

## Introduction

Parkinson’s disease (PD) is one of the most common neurodegenerative diseases, affecting approximately 1% of the population over the age of 60 years (Abbas et al., 2017). The most widely recognized underlying neuropathology for PD is the degeneration of the nigrostriatal dopaminergic projection pathway. Over the past decades, many markers in this pathway have been exploited in targeted imaging approaches, such as the synthesis and presynaptic storage of dopamine (DA), the reuptake of DA with dopamine transporter (DAT), and the expression of the dopamine D2-type receptor (D2R) on the post-synaptic striatal neurons (Frey, 2017).

The degeneration and loss of dopamine neurons in the substantia nigra and striatum of PD patients are accompanied by a decrease in the number and function of DAT in the presynaptic membrane (Piccini, 2003). According to the International Parkinson and Movement Disorder Society Clinical Diagnostic Criteria for Parkinson’s Disease (MDS-PD Criteria), the presence of normal presynaptic DAT on imaging is one way to rule out PD (Postuma et al., 2015). It has been discovered that the uptake of DAT tracers in PD patients’ striatum is significantly lower than that in normal individuals (Marek et al., 2001). DAT imaging can also be applied for the evaluation of severity, progression, and treatment response (Brooks, 2016). The quantitative analysis of DAT function is important for the diagnosis and evaluation of PD.

For the quantification of DAT binding in the striatum in PET imaging, the method of manually drawing region of interest (ROI) on the striatum directly was used; however, automatic procedures for semi-quantification of DAT tracer uptake in the striatum were required for several reasons. Firstly, the conventional ROI method is time-consuming and vulnerable to inter- and intra-operator variability (Tondeur et al., 2010). Secondly, manual and geometric methods usually perform ROI drawing on a single slice or on a sum of adjacent slices and cannot provide information reflecting the 3D complexity of the whole striatal structure. Thirdly, as the radioligand uptake in the posterior putamen is markedly reduced even in the early stage of PD (Nurmi et al., 2003), it is frequently difficult to trace the putaminal margins without reference to magnetic resonance (MR) imaging.

Several automatic quantification methods for DAT imaging evaluation have been released in recent years, such as BasGan (Basal Ganglia, freely available from the Italian association of Nuclear Medicine^1^), DaTView (Nihon Medi-Physics, Tokyo, Japan), DaTQUANT (GE Healthcare, Little Chalfont, United Kingdom), and BRASS (Hermes BRASS software, Nuclear Diagnostics AB, Sweden) (Garibotto et al., 2013; Skanjeti et al., 2015; Yokoyama et al., 2017). Most of them can give the DAT binding values in the basal ganglia automatically. They are based upon a database of healthy controls generated for DAT-SPECT with ^123^I-FP-CIT (^123^I-ioflupane, DaTSCAN, GE Healthcare, Ltd., Little Chalfont, United Kingdom). With statistical parametric mapping (SPM, Wellcome Trust Centre for Neuroimaging, London, United Kingdom) and MATLAB (MathWorks, Natick, MA, United States), some research about quantitative analysis of DAT PET imaging has obtained satisfactory results (Kim et al., 2015). However, the clinical application has been hampered by the complicated data processing, such as conversion of data format, registration, spatial normalization of images, and so on. Furthermore, the abovementioned automatic software cannot analyze ^18^F-fluorodeoxyglucose (^18^F-FDG) PET, which is important for the differential diagnosis of Parkinsonian syndromes, such as multiple system atrophy (MSA), progressive supranuclear palsy (PSP), corticobasal degeneration (CBD), and Lewy body dementia (LBD) (Walker et al., 2018).

NeuroQ (version 3.5, Syntermed, Inc., Atlanta, GA, United States) is a commercial brain imaging analytic software tool that can assist with the regional assessment of human brain scans through automated quantification of mean pixel values lying within 47 standardized regions of interest (S-ROI’s). In this study, we sought to quantitate the PET/CT imaging of ^11^C- 2β-carbomethoxy-3β-(4-fluorophenyl) tropane (^11^C-CFT) and ^18^F-FDG with this software, and to evaluate its potential for diagnosis and assessment of PD.

## Materials and Methods

### Patients

We retrospectively evaluated cases of patients who had undergone both ^11^C-CFT and ^18^F-FDG PET/CT imaging in our center. The inclusion criteria were as follows: (1) fulfilling the diagnosis of PD, MSA, or ET according to the clinical criteria (Gilman et al., 2008; Postuma et al., 2015; Bhatia et al., 2018); (2) an interval between ^11^C-CFT and ^18^F-FDG PET/CT imaging of less than 2 weeks; (3) clinical follow-up at least 6 months after the initial PET/CT imaging without change in diagnosis. Exclusion criteria were evidence of vascular disease on computed tomography (CT) or MRI. Thirty-eight patients were selected, including 20 with PD, 10 with MSA and 8 with essential tremor (ET). At the same time, we recruited 11 volunteers without identifiable neuropsychiatric disease or symptoms as the normal control group (NC).

There were 24 males and 25 females with an average age of 59.16 ± 10.68 years in this study. The severity of motor symptoms of all patients was evaluated by the Hoehn-Yahr (H&Y) staging scale (Martínez-Martín et al., 2015). The PD patients were divided into mild (≤2.5) and moderate-severe (>2.5) disease according to the H&Y scale (Goetz et al., 2004). The study was performed in accordance with the Declaration of Helsinki and approved by the Ethics Committee of Tongji Medical College, Huazhong University of Science and Technology. Patients provided written informed consent.

### Image Acquisition and Reconstruction

Prior to the ^11^C-CFT PET/CT scan, patients were required to undergo a 12-h withdrawal of the medication to avoid the interference to the binding capacity of DAT. Sixty minutes after intravenous injection of ^11^C-CFT (185–370 MBq), a CT and a 15-min PET scan in three-dimensional mode were acquired using a Discovery VCT PET/CT (GE Healthcare, Milwaukee WI, United States). CT acquisition was done with 140 kVp and 200 mAs, which was used for attenuation correction and localization of the lesion site. CT slice thickness was set to the same slice thickness of PET imaging (3.75 mm). For PET imaging, the FORE-Iterative reconstruction algorithm with 20 subsets, 2 iterations, and 2.14 mm (full width at half maximum) post-filtering was used.

^18^F-FDG PET/CT scanning was performed on a different day before or after the ^11^C-CFT PET/CT scanning within 2 weeks. All participants fasted for at least 6 h and stopped any drugs that could affect brain metabolism for at least 12 h before the ^18^F-FDG PET/CT acquisition. An ^18^F-FDG dose of 0.1 mCi/kg (3.7 MBq/kg) was intravenously injected after ensuring the blood glucose level was ≤200 mg/L. The participants rested in a quiet and dimly lit room before and after the ^18^F-FDG injection until the start of imaging. The settings of the image acquisition and data reconstruction were similar to those of the ^11^C-CFT PET/CT scan.

### Data Processing

In the manual measuring method, ROIs in the ^11^C-CFT PET/CT were outlined on the CT fusion images. Three slices including most of the volume of the striatum on CT images were selected, and the caudate nuclei and putamen were outlined on each slice. Mean radioactivity counts of each ROI were calculated independently. Being devoid of DAT, the cerebellum (Cb) was selected as the reference area and the average counts from the three consecutive layers were calculated. The ^11^C-CFT uptake ratio of the caudate nuclei to the cerebellum (CN/Cb) and putamen to cerebellum (Pu/Cb) was expressed as (ROI - Cb)/Cb.

**FIGURE 1:**
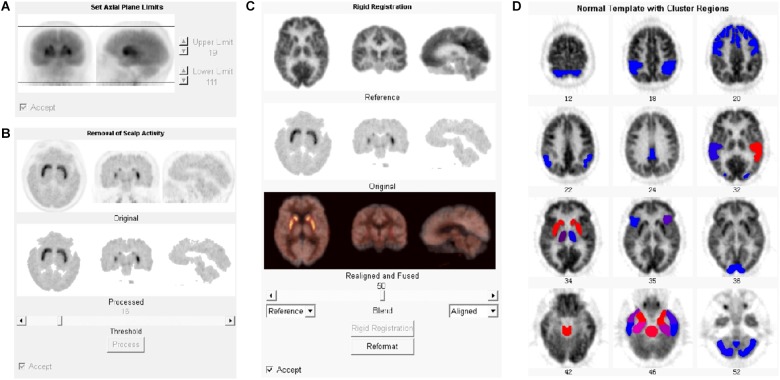
Processing steps for PET data with NeuroQ software. After being imported into NeuroQ, the PET DICOM data were treated with four processing steps: **(A)** setting axial limits, **(B)** removing scalp activity, **(C)** rigid registration, and **(D)** reformatting to fit into the standard template with 47 clusters. For ^18^F-FDG PET data, the results of each ROI could be compared to the normal database and displayed the standard deviation (SD) from mean value of the database with the color scale. Blue to red indicates the increasing of the SD.

^11^C-CFT and the ^18^F-FDG PET files in DICOM format were imported into the NeuroQ software program for automatic quantification. The automatic processing steps included setting axial limits, removing scalp activity, rigid registration, and reformatting (Figure 1). The registration algorithm used for reformatting was a robust spatial transformation method published by Henry Huang and Ed Hoffman (Tai et al., 1997). The subjects’ data were reformatted with 10 iterations to fit into the standard template. The average number of counts per second per pixel for each ROI was calculated. Choosing different references, the value of 47 clusters (ROIs), including caudate nuclei and putamen, could be normalized with the reference. The cerebellum (average value among all pixels in cerebellum regions) was selected as the reference for ^11^C-CFT, while the whole brain (average value among all pixels in the whole brain) was selected for ^18^F-FDG.

### Statistical Analyses

Data for the study variables were expressed as mean ± SD. SPSS21 (IBM, Corp., Armonk, NY, United States) was used for the statistical analysis. Correlations between the manual method and the NeuroQ method were analyzed with the Pearson correlation coefficient. One-way ANOVA analyses were carried out for the different diagnostic groups and the different grades of PD. The difference between the ^11^C-CFT uptake of the symptomatic and asymptomatic side in early asymmetric PD was analyzed with two-sided paired *t*-tests. In addition, to evaluate the diagnostic performance of the NeuroQ software method, we calculated receiver-operating characteristic (ROC) curves. The decision cut-off was considered optimal when the product of paired values for sensitivity and specificity reached its maximum.

## Results

### Correlation Between the Manual Method and NeuroQ Method

**FIGURE 2:**
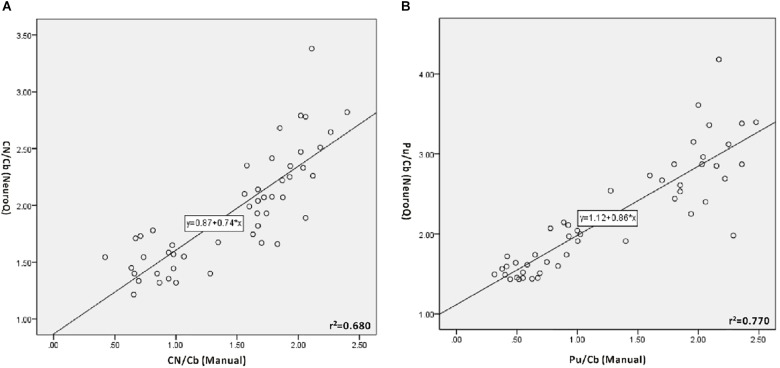
Correlation between manual method and NeuroQ software for the ^11^C-CFT PET analysis. **(A)** Correlation for the uptake ratio of caudate nuclei to the cerebellum (CN/Cb). CN/Cb (Manual) represents the data obtained with the manual method; CN/Cb (NeuroQ) represents the data obtained with NeuroQ software. A good correlation can be seen (*r^2^*= 0.680, *P* < 0.01). **(B)** Correlation for the uptake ratio of putamen to the cerebellum (Pu/Cb). Pu/Cb (Manual) represents the data obtained with manual the method; Pu/Cb (NeuroQ) represents the data obtained with NeuroQ software. There is good correlation between them (*r^2^*= 0.770, *P* < 0.01).

The values of CN/Cb and Pu/Cb were obtained with the manual method and the NeuroQ software. The data of the same ROI from the two methods were analyzed with bivariate correlation analysis (Figure 2 and Table 1). In all participants, there was a significant correlation level, measured as 0.01 (bilateral) between the two methods in both the CN/Cb and Pu/Cb (Figure 2, CN/Cb: *r^2^*= 0.680; Pu/Cb: *r^2^*= 0.770). In different diagnostic groups, the correlation was also significant in all groups except the CN/Cb of MSA and ET group.

**Table 1:** Correlation between the manual method and the NeuroQ method for the ^11^C-CFT uptake ratio of ROIs.

Variables	*r*^2^ of all participants	*r^2^* in different diagnostic groups			
		PD	NC	MSA	ET
CN/Cb (Manual) and CN/Cb (NeuroQ)	0.680^∗^	0.772^∗^	0.421^Δ^	0.381	0.263
Pu/Cb (Manual) and Pu/Cb (NeuroQ)	0.770^∗^	0.712^∗^	0.457^Δ^	0.571^Δ^	0.433^Δ^


*PD, Parkinson’s disease, *n* = 20; NC, normal control, *n* = 11; MSA, multiple system atrophy, *n* = 10; ET, essential tremor, *n* = 8; ^∗^significant correlation at 0.01 level (bilateral); ^Δ^significant correlation at 0.05 level (bilateral).*

### Differential Diagnosis With Data From NeuroQ

One-way ANOVA was performed to analyze the difference of ^11^C-CFT uptake among groups (PD, NC, MSA, and ET). The ^11^C-CFT uptake values of CN/Cb and Pu/Cb are shown in Table 2. The NeuroQ software could automatically obtain CN/Cb and Pu/Cb normalized to the cerebellum. CN/Cb and Pu/Cb were markedly decreased in the PD compared with those of NC (CN/Cb: 1.67 ± 0.36 vs. 2.23 ± 0.36, *P* < 0.01; Pu/Cb: 1.74 ± 0.31 vs. 2.82 ± 0.43, *P* < 0.01) and ET (CN/Cb: 1.67 ± 0.36 vs. 2.50 ± 0.47, *P* < 0.01; Pu/Cb: 1.74 ± 0.31 vs. 3.16 ± 0.49, *P* < 0.01). This outcome could also be found between MSA and NC (CN/Cb: 1.74 ± 0.34 vs. 2.23 ± 0.36, *P* < 0.01; Pu/Cb: 1.79 ± 0.35 vs. 2.82 ± 0.43, *P* < 0.01). However, no statistical difference could be seen between PD and MSA or NC and ET. Similar results were obtained with the manual method.

**Table 2:** Single factor analysis of variance (ANOVA) of ^11^C-CFT and ^18^F-FDG uptake in ROIs between different diagnostic groups.

Test groups	^11^C-CFT				^18^F-FDG				
	Manual		NeuroQ		NeuroQ				
	CN/Cb	Pu/Cb	CN/Cb	Pu/Cb	CN	Pu	MB	Pons	Cb
(1) PD	1.16 ± 0.50	0.69 ± 0.27	1.67 ± 0.36	1.74 ± 0.31	0.93 ± 0.05	1.19 ± 0.06	0.72 ± 0.04	0.65 ± 0.03	0.92 ± 0.04
(2)NC	1.82 ± 0.24	1.98 ± 0.21	2.23 ± 0.36	2.82 ± 0.43	0.96 ± 0.04	1.18 ± 0.04	0.73 ± 0.02	0.66 ± 0.02	0.91 ± 0.03
(3) MSA	1.21 ± 0.52	1.02 ± 0.62	1.74 ± 0.34	1.79 ± 0.35	0.96 ± 0.06	1.15 ± 0.10	0.74 ± 0.02	0.58 ± 0.03	0.71 ± 0.07
(4) ET	2.03 ± 0.21	2.14 ± 0.25	2.50 ± 0.47	3.16 ± 0.49	0.94 ± 0.05	1.18 ± 0.04	0.72 ± 0.02	0.65 ± 0.02	0.90 ± 0.03
Sig.1/2	0.000^∗^	0.000^∗^	0.000^∗^	0.000^∗^	0.067	0.608	0.186	0.302	0.630
Sig.1/3	1.000	0.577	0.644	0.743	0.092	0.064	0.048^Δ^	0.000^∗^	0.000^∗^
Sig.1/4	0.000^∗^	0.000^∗^	0.000^∗^	0.000^∗^	0.521	0.631	0.750	0.911	0.318
Sig.2/3	0.030^Δ^	0.004^∗^	0.004^∗^	0.000^∗^	0.930	0.221	0.520	0.000^∗^	0.000^∗^
Sig.2/4	0.284	0.665	0.125	0.059	0.355	0.985	0.430	0.461	0.607
Sig.3/4	0.004^∗^	0.001^∗^	0.000^∗^	0.000^∗^	0.408	0.266	0.175	0.000^∗^	0.000^∗^


*PD, Parkinson’s disease, *n* = 20; NC, normal control, *n* = 11; MSA, multiple system atrophy, *n* = 10; ET, essential tremor, *n* = 8; Manual, the manual method; NeuroQ, NeuroQ method; CN/Cb, ^*11*^C-CFT uptake ratio of caudate nuclei to cerebellum; Pu/Cb, ^*11*^C-CFT uptake ratio of putamen to cerebellum; CN, ^*18*^F-FDG uptake value of caudate nuclei; Pu, ^*18*^F-FDG uptake value of putamen; MB, ^*18*^F-FDG uptake value of Midbrain; Pons, ^*18*^F-FDG uptake value of Pons; Cb, ^*18*^F-FDG uptake value of cerebellum; ^∗^*P* < 0.01; ^Δ^*P* < 0.05.*

For the ^18^F-FDG PET, we chose CN, Pu, midbrain (MB), pons, and Cb as the ROIs which were important for the differential diagnosis of Parkinsonian syndrome. The values of these ROIs were obtained with the NeuroQ software and normalized with the average value of the whole brain (Table 2). Analyzed with one-way ANOVA, we found that the values of the pons and Cb in the MSA group were significantly lower than those in other groups (Pons: 0.58 ± 0.03; Cb: 0.71 ± 0.07, *P* < 0.01); however, there were nearly no significant difference among groups for the other ROIs.

### Diagnostic Performance of the Data From NeuroQ

We used ROC analysis to evaluate the discriminative power and to determine the diagnostic cutoff of CN/Cb and Pu/Cb with the NeuroQ software. The results are shown in Table 3. For Pu/Cb, the largest area under the curve (AUC) was shown in PD vs. ET (1.000, *P* < 0.01), followed by PD vs. NC (0.982, *P* < 0.01). The cutoff points of Pu/Cb for PD vs. NC and PD vs. ET were 2.20 and 2.62, with high sensitivity and specificity. For PD vs. MSA, the AUC of Pu/Cb was 0.553 (*P* > 0.05), and the largest Youden index was just 0.250. Similar results were obtained from the CN/Cb.

**Table 3:** Receiver-operating characteristic (ROC) curve analysis of ^11^C-CFT and ^18^F-FDG between different diagnostic groups (NeuroQ).

Test groups	Test variable	AUC	Sig.	Youden index	Cutoff	Sensitivity	Specificity
PD vs. NC	CN/Cb	0.866	0.001^∗^	0.700	1.71	70.0%	100%
	Pu/Cb	0.982	0.000^∗^	0.950	2.20	95.0%	100%
PD vs. MSA	CN/Cb	0.593	0.110	0.350	1.56	55.0%	80.0%
	Pu/Cb	0.553	0.107	0.250	1.50	35.0%	90.0%
PD vs. ET	CN/Cb	0.925	0.001^∗^	0.775	2.11	87.5%	90.0%
	Pu/Cb	1.000	0.000^∗^	1.000	2.62	100.0%	100.0%
MSA vs. NC	FDG-Pons	0.991	0.000^∗^	0.909	0.63	90.9%	100.0%
	FDG-Cb	1.000	0.000^∗^	1.000	0.86	100.0%	100.0%
MSA vs. PD	FDG-Pons	0.980	0.021^Δ^	0.850	0.61	90.0%	0.950
	FDG-Cb	1.000	0.000^∗^	1.000	0.85	100.0%	100.0%
MSA vs. ET	FDG-Pons	1.000	0.000^∗^	1.000	0.63	100.0%	100.0%
	FDG-Cb	1.000	0.000^∗^	1.000	0.85	100.0%	100.0%
PD vs. MSA	CN/Cb and FDG-Pons	0.995	0.000^∗^	0.950	NA	95.0%	100.0%
	CN/Cb and FDG-Cb	1.000	0.000^∗^	1.000	NA	100.0%	100.0%
	Pu/Cb and FDG-Pons	1.000	0.000^∗^	1.000	NA	100.0%	100.0%
	Pu/Cb and FDG-Cb	1.000	0.000^∗^	1.000	NA	100.0%	100.0%


*PD, Parkinson’s disease, *n* = 20; NC, normal control, *n* = 11; MSA, multiple system atrophy, *n* = 10; ET, essential tremor, *n* = 8; CN/Cb, ^*11*^C-CFT uptake ratio of caudate nuclei to cerebellum; Pu/Cb, ^*11*^C-CFT uptake ratio of putamen to cerebellum; FDG-Cb, ^*18*^F-FDG uptake value of cerebellum; FDG-Pons, ^*18*^F-FDG uptake value of Pons; AUC, area under the curve; ^∗^*P* < 0.01; ^Δ^*P* < 0.05; NA, not applicable.*

For the ^18^F-FDG PET, both the Pons and Cb showed large AUC in MSA vs. NC (0.991, *P* < 0.01 and 1.000, *P* < 0.01), MSA vs. PD (0.980, *P* < 0.05 and 1.000, *P* < 0.01) and MSA vs. ET (1.000, *P* < 0.01 and 1.000, *P* < 0.01). To differentiate MSA from the other three groups, the cutoff points of FDG uptake value were 0.61–0.63 in pons and 0.85–0.86 in Cb, which possessed high sensitivity and specificity (Table 3).

To distinguish between PD and MSA, we analyzed the discriminative power of the combination of ^11^C-CFT and ^18^F-FDG. Using binary logistic regression, ROC analysis of CN/Cb and FDG-Pons, CN/Cb and FDG-Cb, Pu/Cb and FDG-Pons, as well as Pu/Cb and FDG-Cb was performed (Table 3). Better diagnostic performance was obtained with this combination method (AUC: 0.995–1.000; Youden index: 0.950–1.000; sensitivity: 95.0–100%; specificity: 100%).

### Evaluation of the Severity of PD

In this study, 20 PD patients were divided into mild grade (H&Y ≤ 2.5, *n* = 11) and moderate-severe grade (H&Y > 2.5, *n* = 9). To evaluate the possibility of the NeuroQ method assessing the severity of PD, the uptake of ^11^C-CFT in each ROI was compared among normal controls, mild grade PD and moderate-severe grade PD (Table 4). The results showed that compared to the NC group, Pu/Cb appeared clearly to be decreasing in the mild grade PD (1.92 ± 0.33 vs. 2.82 ± 0.43, *P* < 0.01); however, no significant decrease of CN/Cb was observed until the moderate-severe grade (1.43 ± 0.11 vs. 2.23 ± 0.36, *P* < 0.01). Compared to the mild grade PD, both CN/Cb and Pu/Cb of the moderate-severe grade PD decreased more significantly (CN/Cb: 1.87 ± 0.36 vs. 1.43 ± 0.11, *P* < 0.05; Pu/Cb: 1.92 ± 0.33 vs. 1.53 ± 0.10, *P* < 0.05). Similar results were obtained with the data from the manual method (Table 4).

**Table 4:** Single factor analysis of variance (ANOVA)of ^11^C-CFT uptake in basal ganglia between different clinical grade.

Clinical grade	Age (Y)	Course (Y)	Manual		NeuroQ	
			CN/Cb	Pu/Cb	CN/Cb	Pu/Cb
(1) Mild	59.18 ± 10.91	3.00 ± 0.87	1.50 ± 0.42	0.90 ± 0.19	1.87 ± 0.36	1.92 ± 0.33
(2) Moderate-Severe	57.67 ± 12.14	3.82 ± 2.66	0.75 ± 0.20	0.44 ± 0.08	1.43 ± 0.11	1.53 ± 0.10
(3) NC	61.09 ± 16.40	NA	1.81 ± 0.24	1.98 ± 0.21	2.23 ± 0.36	2.82 ± 0.43
Sig.1/2	0.795	0.928	0.001^∗^	0.005^∗^	0.014^Δ^	0.018^Δ^
Sig.1/3	0.730	NA	0.241	0.000^∗^	0.157	0.000^∗^
Sig.2/3	0.557	NA	0.000^∗^	0.000^∗^	0.000^∗^	0.000^∗^


*Mild grade, H&Y ≤ 2.5, *n* = 11; Moderate-Severe grade, H&Y > 2.5, *n* = 9; NC, normal control, *n* = 11; Manual, manual method; NeuroQ, NeuroQ method; CN/Cb, ^*11*^C-CFT uptake ratio of caudate nuclei to cerebellum; Pu/Cb, ^*11*^C-CFT uptake ratio of putamen to cerebellum; ^∗^*P* < 0.01; ^Δ^*P* < 0.05; NA, not applicable.*

### Evaluating the Asymmetric Change of DAT Function in Early PD

There were 8 PD patients in the early stage with asymmetric motor disorder. Paired *t*-tests were performed to evaluate the difference of the ROI between the symptomatic and asymptomatic side (Table 5). The data obtained from NeuroQ software showed that there was no significant difference for the CN/Cb between the two sides (2.06 ± 0.32 vs. 1.98 ± 0.27, *t* = 1.78, *P* > 0.05). However, the Pu/Cb of asymptomatic side was significantly lower than that of the symptomatic side (2.17 ± 0.30 vs. 1.95 ± 0.22, *t* = 3.98, *P* < 0.01). Similar results were obtained with the data from the manual method.

**Table 5:** Paired *t*-test for the ^11^C-CFT uptake of basal ganglia between the symptomatic side and asymptomatic side in the early asymmetric PD patients.

	Side	Manual		NeruroQ	
		CN/Cb	Pu/Cb	CN/Cb	Pu/Cb
M ± SD	Symptomatic	1.70 ± 0.33	1.06 ± 0.25	2.06 ± 0.32	2.17 ± 0.30
	Asymptomatic	1.65 ± 0.30	0.85 ± 0.10	1.98 ± 0.27	1.95 ± 0.22
*t*-Value		1.450	3.147	1.779	3.981
Sig.		0.190	0.016^Δ^	0.118	0.005^∗^


**n* = 8; Manual, manual method; NeuroQ, NeuroQ method; CN/Cb, ^*11*^C-CFT uptake ratio of caudate nuclei to cerebellum; Pu/Cb, ^*11*^C-CFT uptake ratio of putamen to cerebellum; ^∗^*P* < 0.01; ^Δ^*P* < 0.05.*

## Discussion

The NeuroQ software we tested can aid in the assessment of human brain scans through quantification of mean pixel values lying within S-ROIs. This software is initially focused on analyzing the ^18^F-FDG uptake in different regions. It has been applied in localizing epileptogenic foci, evaluating the severity of cortex impairment, and identifying the metabolic patterns of different neurodegenerative diseases (Torosyan and Silverman, 2012; Kerr et al., 2013; Akdemir et al., 2014).

Many commercial software tools have been applied in the quantitative analysis of dopamine neuro-imaging in the striatum on SPECT or PET. Good correlation between the traditional manual method and the compute-assisted method should be the prerequisite. Heinzel compared the manual method with some commercial software, such as BRASS and TBX, in the analyzing of I-123-IBZM-SPECT (Heinzel et al., 2015). They found fair agreement among the three methods. However, substantial agreement from the inexperienced investigator was not good in the case of the manual method. This might result from the inter- and intra-operator variability of the manual method. Jeong evaluated the feasibility of FP-CIT-PET template-based quantitative analysis in PD, compared with MR-based and manual methods. The results showed excellent correlation between PET-template based software analysis and the manual method (Jeong et al., 2013).

In our study, we used NeuroQ software to obtain quantitative data in ROIs on ^11^C-CFT PET and ^18^F-FDG PET. Those ROIs included the CN and Pu, which were the main pathologic pathway for PD. The results showed that CN/Cb and Pu/Cb provided by the software were consistent with the manual semi-quantitative method (CN: *r^2^* = 0.680; Pu: *r^2^* = 0.770). Furthermore, we evaluated the correlation of two methods in different diagnostic groups, and significant correlation was observed in all groups except the CN/Cb in the MSA and ET groups. These results indicated that the data from the software could be further analyzed for the function of DAT in the basal ganglia. Compared with the manual method, the automatic registration and segmentation of the standard template with the software could avoid subjective deviation and instability. To the best of our knowledge, this is the first study to quantitatively analyze ^11^C-CFT PET with NeuroQ software.

To further explore the utility of NeuroQ in the diagnosis of PD, the ^11^C-CFT uptake values were compared among different groups. The results proved that CN/Cb and Pu/Cb in the PD group were significantly decreased compared with those of the NC and ET groups. It shows high sensitivity and specificity for excluding the diagnosis of PD (95 and 100%) with a cutoff value of 2.2 using Pu/Cb. The result also presents excellent ability for differentiating parkinsonism, for example with ET, which is without dopaminergic cell loss. Other studies obtained the same results. In a six-center study involving 158 PD patients, 27 ET cases and 35 healthy volunteers, DAT-SPECT was visually assessed, and DAT imaging distinguished probable PD from ET with sensitivity and specificity of 95 and 93% (Benamer et al., 2000).

However, DAT imaging is limited in differentiating PD from other causes of parkinsonism due to lack of specificity. In our study, Pu/Cb in the MSA group also decreased and showed no significant difference from the PD group (1.79 ± 0.35 vs. 1.74 ± 0.31). This is also a limitation of mono-modality imaging for the dopaminergic system. Since the theory of parkinsonism-related patterns was proposed by Eidelberg (Eckert et al., 2005), ^18^F-FDG PET has been developed specifically to identify and measure metabolic abnormalities in parkinsonism. Many research showed that FDG PET has a very good ability to differentiate PD, MSA, PSP, and CBD according to the glucose metabolism pattern (Eckert et al., 2005; Huang et al., 2007; Teune et al., 2013; Matthews et al., 2018). The prominent network abnormality detected in PD is characterized by increased pallidothalamic and pontine metabolic activity, associated with reductions in the premotor cortex and parietal associate regions. Patients with multiple system atrophy parkinsonism type (MSA-p) usually have a conspicuous symmetric reduction of metabolism in the putamen, while patients with multiple system atrophy cerebellar type (MSA-c) have prominent hypometabolism in the pons and cerebellum. Patients with PSP demonstrate symmetric ^18^F-FDG hypometabolism in the striatum and in the frontal lobe neocortices, particularly the dorsal prefrontal and mesial frontal aspects. It is easy to identify patients with CBD by their focal and asymmetric hypometabolism. The ^18^F-FDG metabolic pattern was reported to have 92% accuracy in identification of PD, MSA, PSP, and CBD (Eckert et al., 2005). These characteristics have been proved with the analysis by the NeuroQ software (Akdemir et al., 2014). In our study, the ^18^F-FDG uptake in the pons and Cb in the MSA group was significantly reduced compared to the NC, PD, and ET groups. The combination of ^11^C-CFT and ^18^F-FDG improved the discriminative power significantly. There was no marked reduction of ^18^F-FDG uptake in the CN or Pu in the MSA group, which might be due to the small number of MSA-p subtype cases in this study.

Early diagnosis and severity evaluation are very important for both prognostic implications and treatment decisions (Postuma et al., 2015). Related research showed that the anomalous change of DAT function in the basal ganglia happens more sensitively, and DAT imaging potentially provides a biomarker for early diagnosis and objective monitoring of PD progression (Brooks, 2016). In our study, the decrease of Pu/Cb was significant in the mild grade PD, while a marked decrease of CN/Cb was observed only in the moderate-severe grade PD. It is consistent with the pathological development of PD that the neurodegeneration evolves from the substantia nigra pars compacta (SNc), leading to a progressive decrease in the number of dopaminergic presynaptic terminals in the striatum. Following the loss of DAT function in PD could explain the progression of symptoms and provide a potential means of monitoring the efficacy of putative neuroprotective agents (Ravina et al., 2005).

Because the projections from the SNc terminate predominantly within the ipsilateral striatum, idiopathic Parkinson’s disease is characteristically asymmetric at clinical onset. It has been reported that early PD patients showed the most depressed DAT uptake binding in the posterior putamen contralateral to the more clinically affected limb (Nurmi et al., 2003). Liu proposed the anatomical side-to-side effects of presynaptic dopaminergic deficit on the emergence of akinesia and rigidity symptoms at both voxel and regional levels (Liu et al., 2018). For the early asymmetric PD patients in our study, the data from both methods also revealed more significant reduction of Pu/Cb in the asymptomatic side (manual method: *t* = 3.147, *P* < 0.05; software: *t* = 3.918, *P* < 0.01). This finding indicates the detailed information which might contribute to the precise evaluation for PD.

As a pilot study, some limitations deserve mention. First, the number of cases in each group was limited. In the future, more cases and volunteers will be recruited for further study. To obtain more accuracy and rational analysis, the NC database should be set up and refined according to age and gender. Second, except for the manual semi-quantitative method, the results should be compared with other widely used software, such as BasGan, DaTView, DaTQUANT, BRASS, SPM (statistical parametric mapping) or PMOD. As a source-free and modality-unlimited software, SPM will be the method compared with NeuroQ in our future study. Last but not least, the template in NeuroQ was divided into 47 clusters without detailed subregions for the striatum, and this might limit the accuracy of analysis. If more precise quantitation is needed, a more detailed template should be set in the software.

## Conclusion

NeuroQ software, a commercial software commonly used for ^18^F-FDG PET, could also be applied to analyze the images of ^11^C-CFT PET. The results showed that the quantitative data obtained by the software seemed more accurate and stable, which may provide a novel quantitative method for the differential diagnosis and severity evaluation in parkinsonism and PD.

## Author Contributions

XL substantially contributed to the conception and design, analyzed and interpreted the data, and revised the manuscript critically for important intellectual content. XS acquired, analyzed and interpreted most of the data and drafted the article. FL acquired and analyzed some data. QL and YG prepared the compounds ^11^C-CFT and ^18^F-FDG. WR, FH, and XT acquired the PET images. DW revised the manuscript. All authors read and approved the final manuscript.

## Conflict of Interest Statement

The authors declare that the research was conducted in the absence of any commercial or financial relationships that could be construed as a potential conflict of interest.

## Footnotes

1 https://www.aimn.it/site/index/gdssegretari/
